# Dermal Phospho-Alpha-Synuclein Deposition in Patients With Parkinson's Disease and Mutation of the Glucocerebrosidase Gene

**DOI:** 10.3389/fneur.2018.01094

**Published:** 2018-12-17

**Authors:** Kathrin Doppler, Kathrin Brockmann, Annahita Sedghi, Isabel Wurster, Jens Volkmann, Wolfgang H. Oertel, Claudia Sommer

**Affiliations:** ^1^Department of Neurology, University Hospital Würzburg, Würzburg, Germany; ^2^Department of Neurology, University Hospital Tübingen, Tubingen, Germany; ^3^Department of Neurology, University Hospital Marburg, Marburg, Germany

**Keywords:** Parkinson's disease, glucocerebrosidase mutation, alpha-synuclein, skin biopsy, biomarker

## Abstract

Heterozygous mutations in the glucocerebrosidase gene (*GBA1*) represent the most common genetic risk factor for Parkinson's disease (PD) and are histopathologically associated with a widespread load of alpha-synuclein in the brain. Therefore, PD patients with *GBA1* mutations are a cohort of high interest for clinical trials on disease-modifying therapies targeting alpha-synuclein. There is evidence that detection of phospho-alpha-synuclein (p-syn) in dermal nerve fibers might be a biomarker for the histopathological identification of PD patients even at premotor or very early stages of disease. It is so far unknown whether dermal p-syn deposition can also be found in PD patients with *GBA1* mutations and may serve as a biomarker for PD in these patients. Skin biopsies of 10 PD patients with different *GBA1* mutations (six N370S, three E326K, one L444P) were analyzed by double-immunofluorescence labeling with anti-p-syn and anti-protein gene product 9.5 (PGP9.5, axonal marker) to detect intraaxonal p-syn deposition. Four biopsy sites (distal, proximal leg, paravertebral Th10, and C7) per patient were studied. P-syn was found in six patients (three N370S, three E326K). P-syn deposition was mainly detected in autonomic nerve fibers, but also in somatosensory fibers and was not restricted to a certain *GBA1* mutation. In summary, dermal p-syn in PD patients with *GBA1* mutations seems to offer a similar distribution and frequency as observed in patients without a known mutation. Skin biopsy may be suitable to study p-syn deposition in these patients or even to identify premotor patients with *GBA1* mutations.

## Introduction

Pre-mortal diagnosis of Parkinson's disease (PD) is based on its clinical presentation with tremor, rigor, akinesia, and postural instability. Alpha-synuclein aggregates in neurons of the substantia nigra represent the histopathological hallmark of the disease and are not only considered as post-mortem disease marker but also offer insights into the pathogenesis of the disease.

In the last few years, focus has been set on the onset of PD-associated neurodegeneration and it is known that the disease starts many years before the onset of motor symptoms (1). Non-motor symptoms such as obstipation, hyposmia, depression, or rapid eye movement sleep behavior disorder (RBD) may occur during the prodromal phase of PD when the patients do not show any motor symptoms but alpha-synuclein deposition and neuronal loss can already be found in the brain (2). Major efforts of drug development focus on the deposition of alpha-synuclein as a probable pathogenic key event. Clinical trials of drugs targeting alpha-synuclein deposition require reliable identification of patients with primarily alpha-synuclein-driven neurodegeneration who are in the prodromal stage of the disease and in whom pre-mortem non-invasive monitoring of alpha-synuclein deposition is possible. Within a high-risk cohort for PD, skin biopsy might be a potential tool to identify individuals at the earliest stages of the disease and to monitor progression of alpha-synuclein deposition. One of such a high-risk PD cohort are patients with RBD (3) and it has already been shown that p-syn deposition can be found in skin biopsies of RBD patients, rendering skin biopsy a potential biomarker for prodromal PD (4, 5). Another risk factor for the development of PD are glucocerebrosidase gene (*GBA1)* mutations that are supposed to be found in 4–10% of all PD patients (6–8) and increase the risk of developing PD 20-fold (8). PD patients carrying *GBA1* mutations are of special interest as a first clinical trial with a substrate reduction inhibitor, GZ/SAR402671, has already started in in this subgroup of PD. However, alpha-synuclein deposition in skin biopsy has not yet been tested in PD patients with *GBA1* mutations.

In the present study, we aimed to evaluate the use of skin biopsy for the detection of p- syn in PD patients with *GBA1* mutations and to evaluate potential differences of dermal p- syn deposition in patients with *GBA1* mutation associated PD compared to results from former studies on patients with idiopathic PD.

## Materials and Methods

### Patients

Ten patients with a known *GBA1* mutation were prospectively recruited at the University Hospital Tübingen (mean age 61.7 (±8.1) years. Initially, they had been recruited for the prospective observational MiGAP study (Markers in *GBA1* associated Parkinson) funded by the DZNE (German Centre for Neurodegenerative Diseases, Site Tuebingen)^1^ Out of 100 patients of the MIGAP study, we randomly selected and asked 10 PD patients to take part in the present sub-study. Diagnosis of PD was based on the UK brain bank criteria (9). Stage of disease was assessed using the Hoehn&Yahr scale (10), motor function was evaluated by Unified Parkinson's Disease Ranking Scale part III (UPDRS-III) (11). The bradykinesia score and annual UPDRSIII progression were calculated as previously described (12). Ten age and gender matched healthy controls who were recruited for former studies (5) and whose biopsy material was stored at our department were also investigated. All patients and controls gave oral and written informed consent to participate. The study was approved by the Ethic's committee of the University of Würzburg. Demographic data of all patients and controls and the type of mutation are summarized in Table 1.

**Table 1:** Demographic data and p-syn deposition of patients with *GBA1* mutation.

**Patient no**.	**Duration of disease (y)**	**H&Ystage**	**UPDRS part III**	**Annual UPDRS III progression**	**Bradykinesia score**	**Subtype**	***GBA1* mut**.	**p-syn pos biopsy site**	**p-syn pos structures**	**No. of positive struct**.
1	14	3	70	5	1.7	Equivalent	N370S	LL	ves, db, ep	3
2	6	2	48	8	1.5	Equivalent	N370S	–		0
3	17	2	34	2	0.6	Equivalent	N370S	LL, Th10	subepi, intraepi,ves, db	4
4	6	2.5	35	5.8	1.7	Equivalent	N370S	–		0
5	5	2	32	6.4	1.1	Equivalent	N370S	UL, Th10	db	2
6	1	2	10	10	0.5	Tremor dominant	L444P	–		0
7	8	3	49	6.1	2.5	Equivalent	E326K	UL, LL, C7	db, ves, ep	4
8	14	2	32	2.3	1.6	Equivalent	E326K	UL	subepi	1
9	14	3	53	3.8	2.6	Equivalent	E326K	UL, LL, C7	db, sg	4
10	2	2	36	18	1.8	Acinetic-rigid	N370S	–		0

*No., number; y, years; H&Y, Hoehn&Yahr; GBA1, glucocerebrosidase gene; p-syn, phospho-alpha-synuclein; pos, positive; struct., structures; LL, lower leg; UL, upper leg; ves, vessel; db, dermal nerve bundle; ep, erector pilorum muscle; subepi, subepidermal; intraepi, intraepidermal; sg, sweat gland; UPDRS, Unified Parkinson Disease Rating Scale*.

### Skin Biopsy

Skin punch biopsies were taken from the distal and proximal leg, back (Th10), and neck (C7), fixed with paraformaldehyde and cryconserved until use as previously described (13). Twenty micrometer serial cryosections were cut. Double-immunofluorescence-labeling was performed using anti-PGP9.5 (axonal marker, Zytomed Systems, Berlin, Germany, 1:200) and anti-p-syn (Biolegend, San Diego, CA, United States, 1:500) and appropriate Cy3 and AlexaFluor488-conjugated secondary antibodies (Dianova, Hamburg, Germany, 1:100/1:400).

### Microscopy

Double-immunofluorescence-labeling was assessed in a blinded manner using a fluorescence microscope with CARVII system (Ax10, Zeiss, Oberkochen, Germany/Visitron GmbH, Puchheim, Germany). All slides were scanned for p-syn-positive dermal nerve fibers. Nerve fibers were identified by staining with anti-PGP9.5 and only p-syn deposition within nerve fibers was considered “positive.” A biopsy was assessed “positive” if at least one dermal nerve fiber was immunoreactive for p-syn. P-syn-positive nerve fibers were categorized as sudomotor, vasomotor, pilomotor, or somatosensory (subepidermal plexus or intraepidermal) according to their location. Nerve fibers that could not be assigned to a certain skin structure were assessed as dermal nerve bundles. P-syn deposition was quantified as the number of skin structures that contained at least one p-syn-positive nerve fiber.

### Statistical Evaluation

Statistical analysis was calculated using SPSS Statistics 23 software (IBM, New York, United States). Two-sided Pearson's correlation test was used for correlation analysis. A significance level of 5% was applied.

## Results

**Figure 1:**
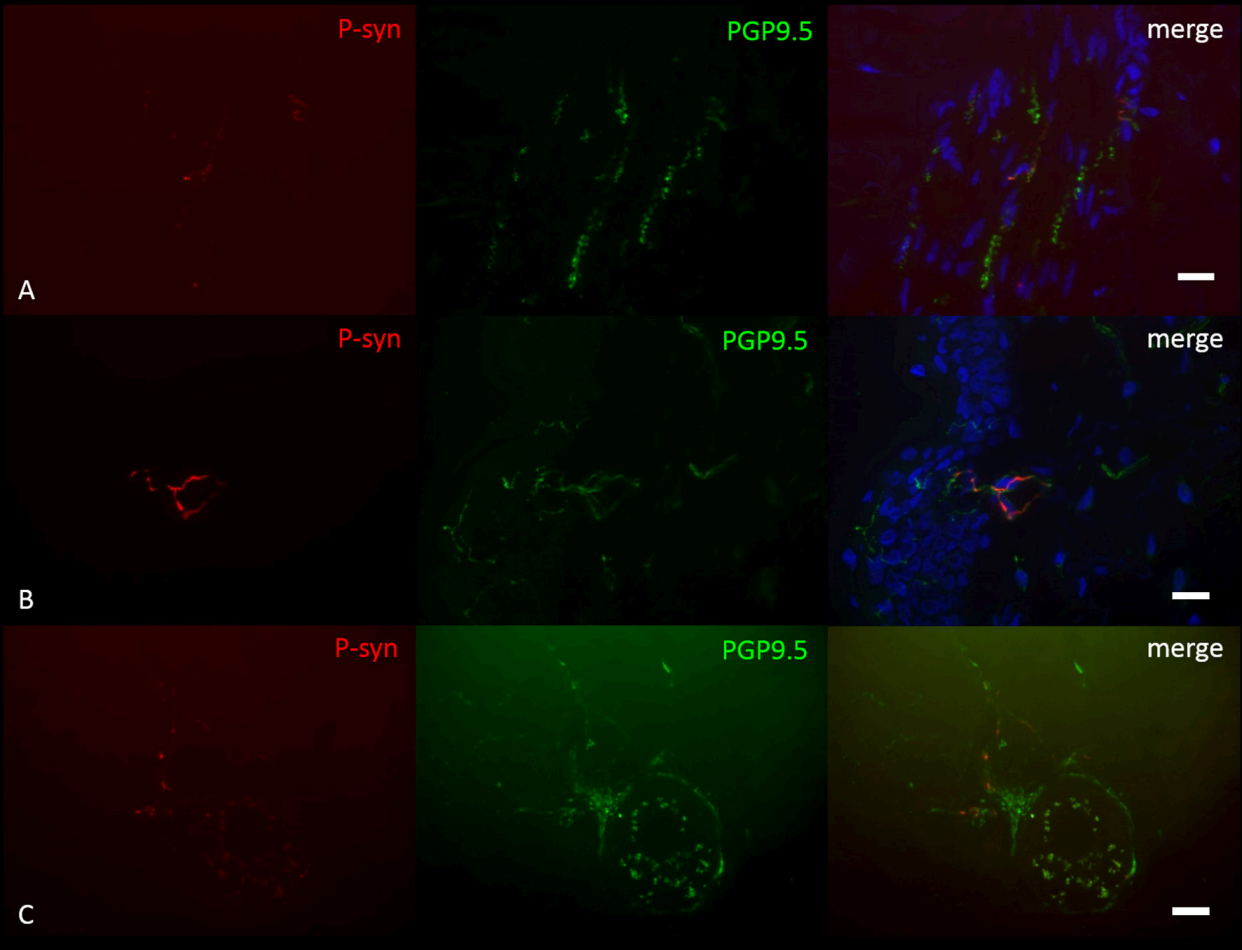
Photomicrographs of a double-immunofluorescence staining with anti-p-syn (red) and anti-PGP9.5 (green). Cell nuclei are stained with DAPI (blue). P-syn deposition is detectable in pilomotor fibers **(A)**, intraepidermal fibers **(B)** and sudomotor fibers **(C)** of patients with *GBA1* mutation-associated PD. Scale Bar = 10 μm.

P-syn deposition was found in 6/10 PD patients with *GBA1* mutations, not in any healthy control (Figure 1). P-syn-positive nerve fibers were found in four biopsies of the distal leg, four of the proximal leg, two of the back, and two of the neck. Autonomic vasomotor fibers were affected in three cases, sudomotor fibers in one patient (Figure 1C), pilomotor in two (Figure 1A), and dermal nerve bundles in five cases. Somatosensory nerve fibers of the subepidermal plexus were found positive in two patients, in one of them, intraepidermal fibers were positive (Figure 1B). P-syn-positive fibers were not restricted to a certain mutation within *GBA1* and were found in 3/6 patients with N370S mutation, 3/3 patients with E326K mutation, and 0/1 patient with L444P mutation. The number of p-syn positive dermal structures correlated with the duration of disease (*p* = 0.02, *r* = 0.71), but not with age at assessment. Correlation analysis between p-syn-positive structures and H&Y stage, bradykinesia score and annual UPDRSIII progression was not significant (H&Y: *p* = 0.06, r = 0.61, bradykinesia score: *p* = 0.37, *r* = 0.32, annual UPDRSIII progression: *p* = 0.08, *r* = −0.58).

## Discussion

Here, we report p-syn deposition in dermal nerve fibers of PD patients carrying a mutation in *GBA1*.

The frequency of 60% in our study is comparable with former skin biopsy studies in idiopathic PD using similar protocols (5, 13, 14). Predominant autonomic involvement with vasomotor fibers as the mostly affected fibers is also in line with previous studies (13, 15). Our results indicate that dermal p-syn pathology of patients with *GBA1* mutations does not differ from idiopathic PD. This corresponds to findings from brain autopsy studies that also did not show a clear difference except for some studies describing more extensive diffuse cortical Lewy bodies (6, 16) that could not be confirmed by others (17, 18). Dermal p-syn deposition was not restricted to a certain mutation and was also found in patients with the E326K mutation which is considered a rather “mild” mutation (19). Our results indicate that clinical and neuropathological similarity between patients with *GBA1* mutations and without can be extended to the PNS, rendering skin biopsy a pre-mortem tool to investigate p-syn pathology in this patient group.

In recent studies no correlation between p-syn deposition and stage or duration of disease could be determined for PD (13, 15). Only in RBD, a potential prodromal stage of PD, a correlation between dermal p-syn and disease progression markers could be found, indicating a steady-state of dermal p-syn deposition during motor stages of disease. In the present study, p-syn positive structures correlated with duration of disease. This might indicate progressive p-syn deposition during the course of disease in patients with *GBA1* mutations. This is of special interest as this subgroup of PD patients was shown to present a more rapid disease progression (20). The frequency of p-syn deposition in early or even prodromal stages of *GBA1* associated PD needs to be investigated in future studies.

The exact underlying pathomechanism of p-syn deposition in patients with *GBA1* mutations is still unclear, but there is evidence that impaired lysosomal function and endoplasmatic reticulum stress play a role (21) and that accumulation of alpha synuclein is promoted by glucocerebrosidase deficiency (22). Involvement of dermal nerve fibers in p-syn pathology in *GBA1* mutation associated PD provides the opportunity for the use of skin biopsy as a pre-mortem easily accessible tissue for the investigation of p-syn pathology in this subgroup of PD.

In summary, this pilot study gives evidence that dermal nerve fibers are affected by p-syn pathology in PD with *GBA1* mutations. A major limitation is the small sample size that does not allow a clear conclusion on the frequency and distribution of p-syn deposition in this subgroup compared to idiopathic PD. Detection of p-syn in skin biopsies of *GBA1*-associated PD is a basic prerequisite for future studies on prodromal *GBA1*-associated PD.

## Author Contributions

KD planned and designed the study, performed, and analyzed skin biopsies and wrote the first draft of the manuscript. KB planned and designed the study, recruited and characterized patients, performed skin biopsies, and revised the manuscript. AS performed and analyzed skin biopsies. IW recruited and characterized patients. JV and WO were involved in the study design. CS was involved in study design and revised the manuscript.

### Conflict of Interest Statement

The authors declare that the research was conducted in the absence of any commercial or financial relationships that could be construed as a potential conflict of interest.

## Footnotes

^1^https://www.dzne.de/forschung/studien/klinische-studien/migap/

## Abbreviations

*GBA1*: glucocerebrosidase gene
PD: Parkinson's disease
RBD: REM sleep behavior disorder
PGP9. 5: protein gene product 9.5
p-syn: phospho-alpha-synuclein.
